# A highly efficient murine model of experimental myopia

**DOI:** 10.1038/s41598-018-20272-w

**Published:** 2018-02-01

**Authors:** Xiaoyan Jiang, Toshihide Kurihara, Hiromitsu Kunimi, Maki Miyauchi, Shin-ichi Ikeda, Kiwako Mori, Kinya Tsubota, Hidemasa Torii, Kazuo Tsubota

**Affiliations:** 1 Laboratory of Photobiology, Keio University School of Medicine, Tokyo, Japan; 2 Department of Ophthalmology, Keio University School of Medicine, Tokyo, Japan

## Abstract

Despite the global pandemic of myopia, the precise molecular mechanism of the onset of myopia remains largely unknown. This is partially because of the lack of efficient murine myopic models that allow genetic manipulation at low cost. Here we report a highly practical and reproducible lens-induced myopia model by specially designed frames and lenses for mice. A lens power dependent myopic induction in mice was shown until minus 30 diopter lenses. The phenotype was significantly stronger than form-deprivation myopia. We presented the protocol for precise evaluations of the state of myopia, including refraction, corneal curvature and axial length using up-to-date devices. We also found that myopic mouse eyes showed decreased visual acuity on optokinetic response examination. Finally, we confirmed the anti-myopic effect of 1% atropine using this model, which showed its potential in drug screening. The strong phenotype, stable evaluation and the potential for gene manipulation utilizing the presented method in mice will accelerate the translational research of myopia.

## Introduction

The global increase of myopia, or short-sightedness, is becoming a serious health hazard in the world^1,2^. Myopia is characterized as elongation of the axial length (AL) of the eyeball; however, the mechanisms of the onset and the progression of AL elongation are largely unknown and further study is needed to develop effective ways for controlling myopia.

The research on myopia has been growing exponentially since the first report of experimental myopia, which was achieved on monkeys by lid fusion^3^. The visual deprivation in young age resulted in a myopic shift in refractive state and an elongation in AL. This phenomenon was soon reproduced on tree shrews^4^, marmosets^5^, mice^6^, and chicks^7^. Besides the form-deprivation myopia (FDM) models mentioned above, myopia can also be induced using minus lenses (lens-induced myopia, LIM) in these species^8–12^. Among them, some types of species especially diurnal animals such as chicks and tree shrews have been used for myopia research frequently since their phenotypes of experimental myopia are greater than others^4,13^. To investigate the molecular mechanism of disease pathogenesis, it is desirable to induce axial elongation in mice with an efficient and effective method in terms of genetic manipulation.

Previous reports by other groups have shown that LIM is inducible in mice^9,14,15^. FDM mouse models have been recognized as relatively robust and widely used while LIM mouse models are considered to be unstable because of the difficulty of fixing the lens in front of the mouse eye^9,14,16–18^. However, form-deprivation leads to relatively weaker myopic phenotypes in mice than minus lenses, especially for AL which sometimes has no statistical significance^9,14,19^. Another merit of LIM is the transparency of lenses to light. Since more and more findings suggest an important role of the light environment on the onset of myopia, experimental treatments such as light exposure have been conducted frequently in recent years^13,20–26^. LIM models would be more suitable for these light-related experiments since the light can pass through the lens without changing its irradiance or wavelength properties. Thus, a refined LIM mouse model is needed.

Besides the difficulty of fixing the lens, another problem for using mice is the small changes in AL. Frozen sections^27^, A-scan ultrasonography^28^, MRI^29^ and many other methods have been used for the measurement of AL of the mouse eye, while the resolutions of these measurements may not be high enough for detecting the small change of the mouse eye after myopia inducement, and the variety of measured values makes it hard to compare results across different laboratories. According to the optical structure of mouse eyes, myopic refractive change of 1 diopter (D) is equivalent to only 5.4–6.5 μm elongation of the AL in mice^30^. Therefore, high resolution measurements are needed for the evaluation of the mouse eye elongation^31^.

Here we show a highly reproducible murine lens-induced myopia model with a newly designed skull-mounted eyeglass using a three-dimensional printer and customized lenses referring to previous reports describing head-mounted goggles^18,32^. The phenotype of myopia was evaluated with a large focal depth spectral-domain optical coherent tomography (SD-OCT) designed for mice. A comparison with a measurement using a μCT system specialized for small animals was also examined. We found that the presented LIM model showed a more significant phenotype than FDM. Besides refraction and AL, we performed an optokinetic response (OKR) examination and found decreased responses on myopic eyes, while the electroretinography (ERG) was not changed, which indicated that these murine myopic eyes have lower visual acuity^33^. Furthermore, a therapeutic effect of topical atropine treatment was confirmed in the model as shown in clinical trials^34,35^. We believe that the current proposed murine model will accelerate the translational research to prevent the myopia onset and progression.

## Results

### Design and the setup method of eyeglasses for mice

**Figure 1:**
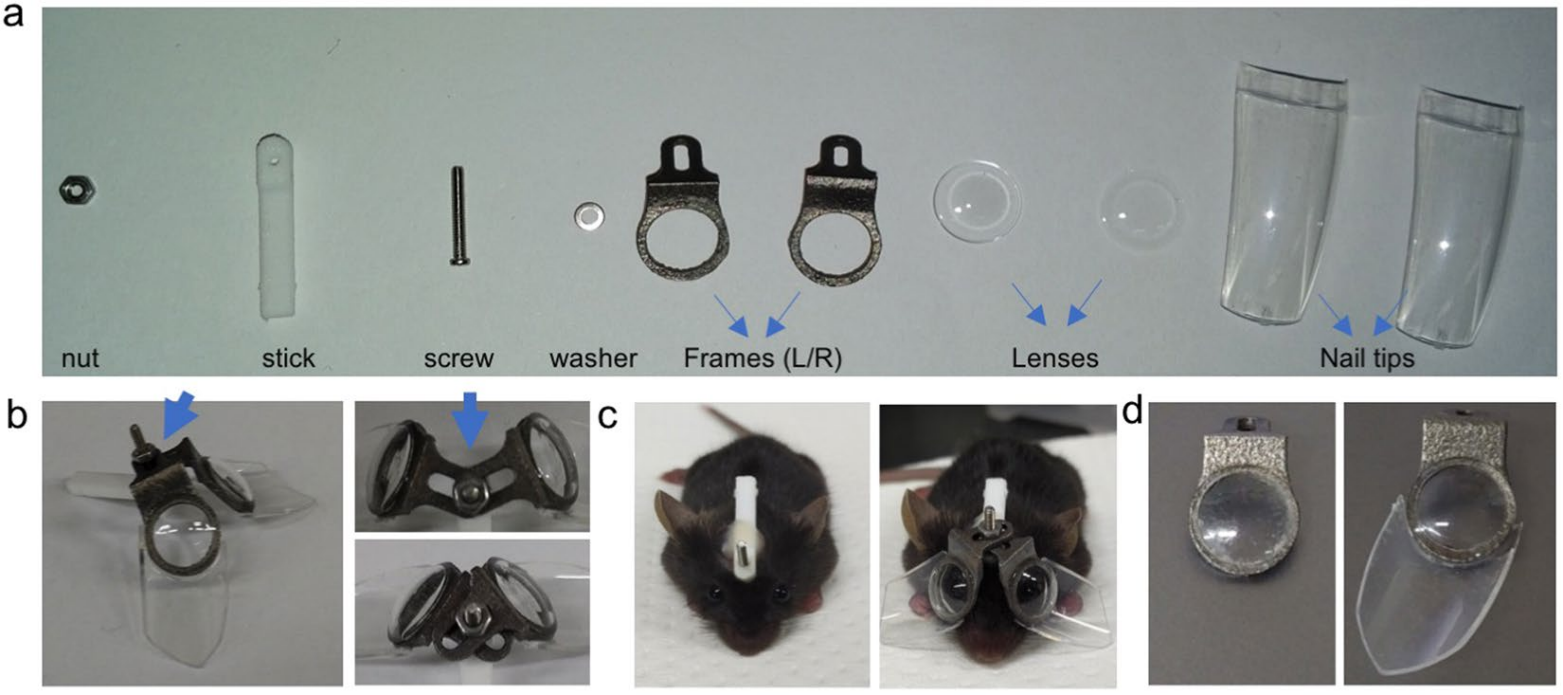
The design of the skull-mounted eyeglass. (**a**) All the parts needed for assembling the mouse glasses. (**b**) An example of assembled glasses for mice. The joint part (blue arrow) allows the left and right frame to be adjusted according to the shape of the mouse skull. (**c**) By loosening the nut, the lens can be removed together with the frame for cleaning. (**d**) Artificial clear fingernail tips are added to prevent the mouse from scratching the lens. Left photo shows the lens worn for three weeks without the protection of the nail tip. Countless scratches can be seen on the surface of the lenses which may influence the input of the vision. No scratches are apparent on the lenses with the protection of the nail tip after three weeks of use.

Frames of the eyeglasses for the mouse LIM were designed according to the shape of the mouse head. All necessary parts were shown in Fig. 1a. The stick and frame were made from nylon and titanium respectively using a three - dimensional printer. The lenses with the power ranging from −50 D to +5 D were made specifically for mice from human hard contact lenses. Lenses were fixed on the frame with cyanoacrylate adhesive. The joint part allows the adjustment of the angle and width of two pieces of the frames to ensure the eye is at the middle of the lens during the experiments (Fig. 1b). The lens can be removed together with the frame for cleaning by loosening the nut (Fig. 1c). Artificial fingernail tips were fixed around the frame to protect the lens from being scratched by the mouse to avoid the use of the Elizabeth collar (Fig. 1d). Trials and errors of the design were shown in Supplementary Fig. 1. Interestingly, we found the angle of the nail tips influenced the outcome of inducement dramatically. When we tested 10 mice with the nail tips vertical to the frame, no significant change in AL was observed in −30 D lens wearing eyes compared with their fellow eyes (4^th^ generation in Supplementary Fig. 1). Therefore, we enlarged the angle of the nail tip (5^th^ generation in Supplementary Fig. 1). This also partially proved that mice can compensate the −30 D lens rather than recognize it as a diffuser. Except for the FDM experiment, all the following experiments in this article were performed using this version.

The surgery for mounting the frame on the mouse head was safe and the self-cure dental adhesive system bonded the frame to the skull stably although some practices were needed. No mice died because of the lens attachment and no dislodge of the frame was found during all the experiments in this study.

### Stable and repeatable measurements of the parameter of the mouse eye

**Figure 2:**
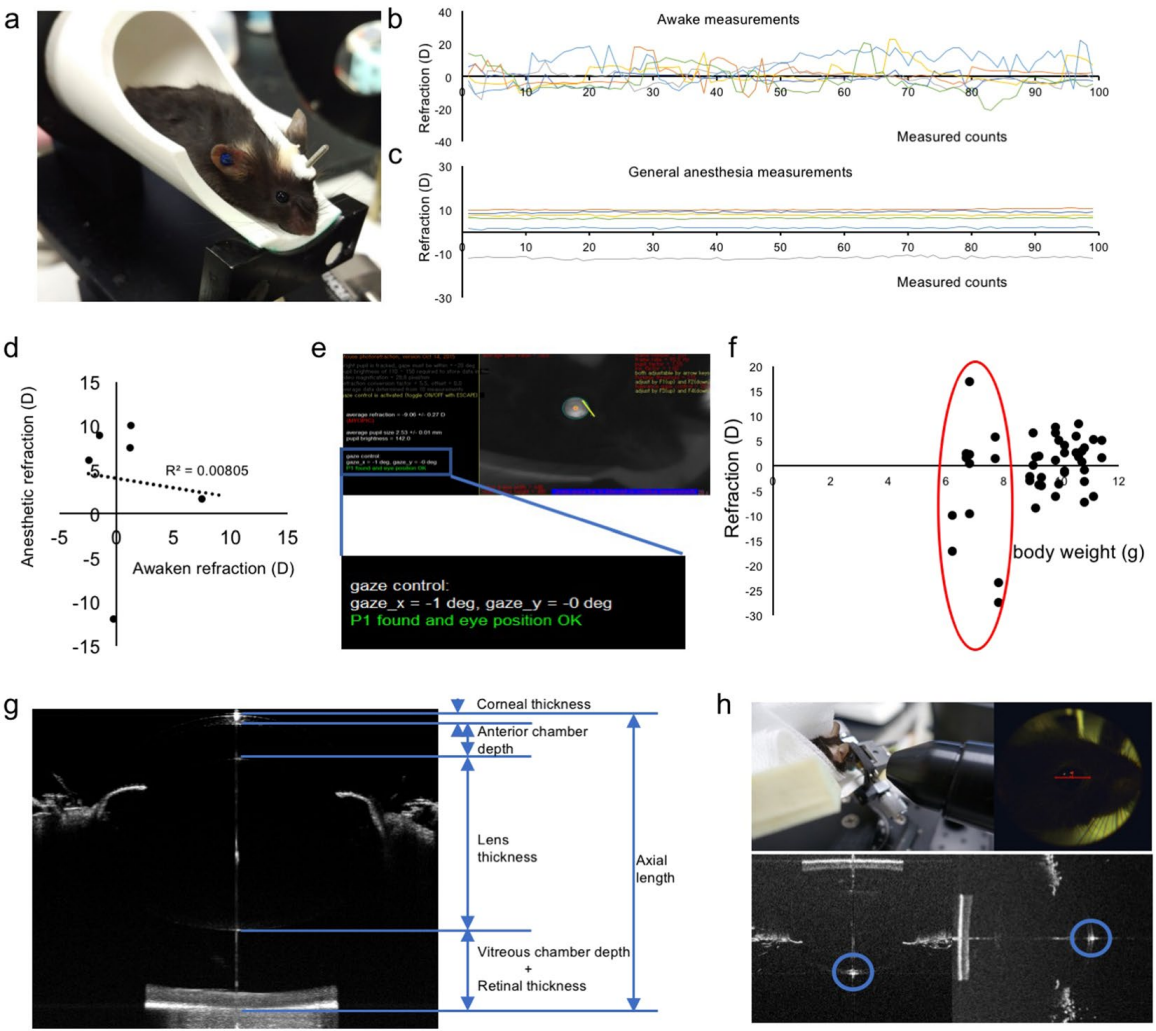
Protocols for stable and repeatable measurements of the parameter of the mouse eye. (**a**) The scene of refraction measurement. To achieve a highly stable result, the mouse is put in an adjustable tube under general anesthesia. The refraction value measured in mice awake (**b**), and under general anesthesia (**c**) is shown. Values are more stable with mice under general anesthesia (n = 6). (**d**) Little correlation between awake and under general anesthesia can be found in the average of 99 times measured values. (**e**) The relative position between the mouse eye and the camera is settled along the optical axis with the gaze control under + /−3 degrees (blue frame). (**f**) The start line of refraction measurements from 23 mice (46 eyes), p21. A strong variation can be observed in eyes from the mice less than 8 grams (red circle). (**g**) The ocular parameters measured using SD-OCT. Since the boundary of the retina in vitreous side becomes hard to recognize near the optic pupil because of the hyaloid vessel, we define the AL as the length between the cornel vertex to the outer boundary of the retina. (**h**) The scene of AL measurement. The AL is measured from the corneal vertex to the half diameter of the optic papilla away from the center. The position of corneal vertex is judged by the point-like reflected light on the cornea (blue circles). The judgement of the boundary of the retina side is shown in Supplementary Fig. 4.

An infrared photorefractor was used to measure the refractive state (Fig. 2a). Six mice were measured during an awake state, and measurements were performed on the same group of mice under general anesthesia with a combination of midazolam, medetomidine and butorphanol tartrate (MMB). We found that the measured values in the awake state were very unstable (Fig. 2b) compared to under general anesthesia (Fig. 2c), and the values taken with these two different conditions had little correlativity (Fig. 2d). According to the reported protocol for infrared photorefractor, the refraction can be obtained with high accuracy as Purkinje images in the middle of the pupil when the gaze control of the software comes close to 0 degree (Fig. 2e)^6^. For some mice, only 4 degrees away from the middle of the pupil resulted in more than 30 diopters’ change in the refraction measured value (Supplementary Fig. 2). Thus, we determined that measuring the mouse under general anesthesia was required with a tolerance gaze control under 3 degrees. To achieve this precise measurement, the tube of the SD-OCT system might be helpful when tweaking the direction of the mouse eye (Supplementary Fig. 3).

It is necessary to mention that a strong variation in refraction existed even among the mice of the same age. This might have resulted from different growth states. The refraction and body weight of 23 mice (46 eyes) at the age of postnatal day 21 (p21) were measured and the results were plotted. We found that the refraction tended to be unstable and extreme hyperopia or myopia appeared in the mice less than 8 grams of body weight (Fig. 2f). To achieve reasonable results after three weeks of induction, using mice with the body weight over 8 grams was appropriate.

The AL of the eye was analyzed by the SD-OCT system tuned for mice (Fig. 2g,h). Although the system had a high resolution of 2.6 μm, precise and repeatable measurements were required to detect the small change of the AL in mice. The definition of AL and other ocular parameters were shown in Fig. 2g. The SD-OCT slice was taken from the corneal vertex to the retina closed to the optical nerve. The boundary of the vitreous body side of retina tends to be hazy near the optical nerve; therefore, the vitreous chamber and retina were measured together. The corneal vertex was judged by the point-like reflected light on the cornea (Fig. 2h). The method to judge where to cut the optical nerve side was shown in Supplementary Fig. 4.

### Comparison of the AL of mice measured using a μCT system

**Figure 3:**
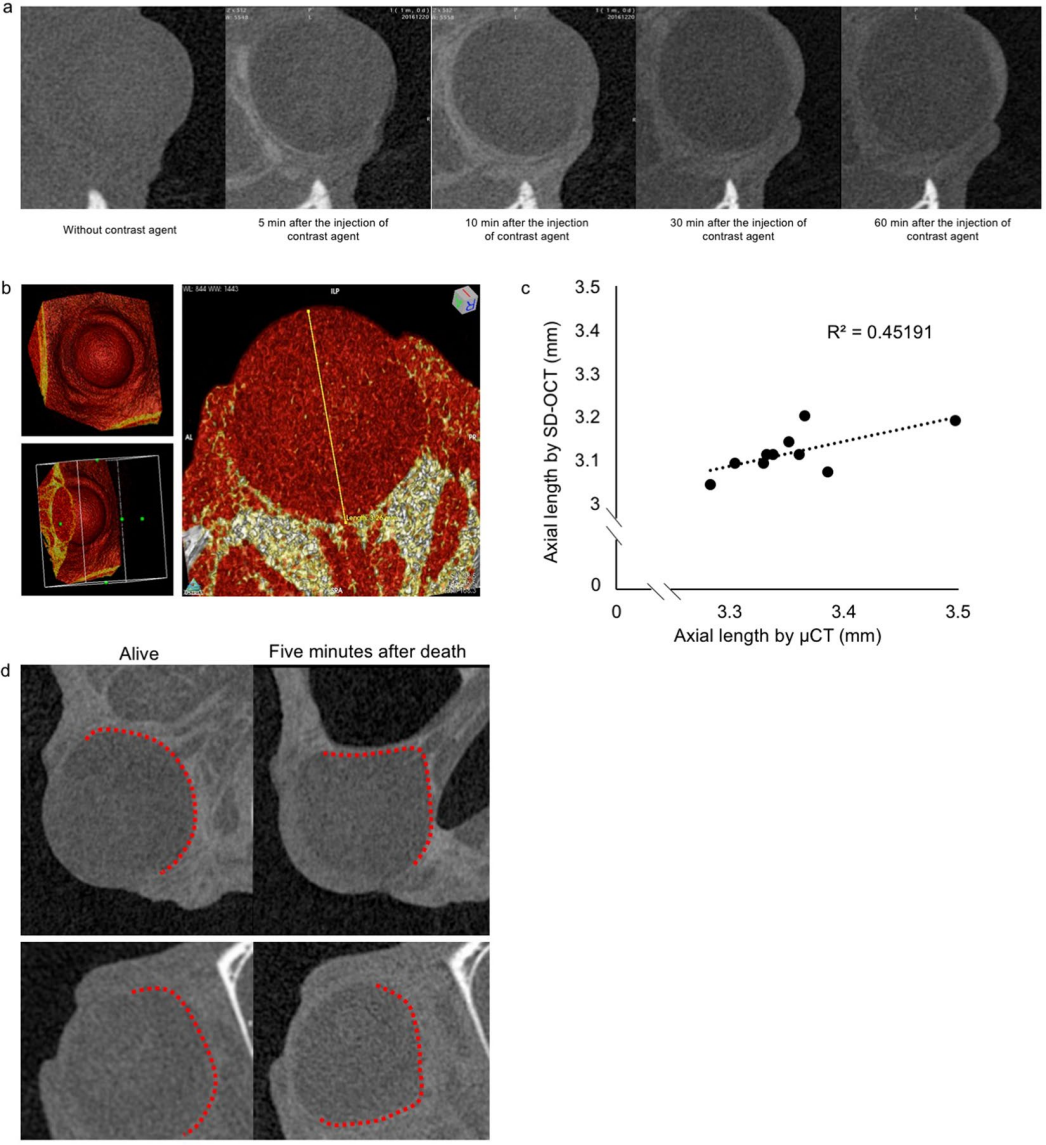
Comparison with the AL of mice measured using μCT. (**a**) An example of the μCT scan of one mouse eye before and after 5 min, 10 min, 30 min, and 60 min of the intravenous injection of the contrast agent, respectively. A time dependent upgrade of contrast can be observed in 10 minutes. We choose 10 min as the proper duration between the injection and the μCT scan. (**b**) An example of three‐dimensional reconstruction of the scan of the eyeball with the contrast agent. The AL is measured from the vertex of the cornea to the optic nerve. (**c**) The relevant curve of ALs measured by μCT and SD-OCT (n = 10). (**d**) The deformation of the eyeball after the mouse dies is detected by μCT scan. By slicing the eyeball at the same position, the obvious shrinkage can be seen only 5 min after the mouse’s death. The eyeball of two separate mice are shown here as examples. Red dots show the boundary of the eye ball.

We further compared the AL values measured by the OCT system to those by a μCT system for small animal use. By injecting iohexol for contrast enhancement, the borderline of the eye began to be seen five minutes after the injection, and the enhancement reached the peak around 10 min (Fig. 3a). The whole eyeball can be seen after three-dimensional reconstruction, and the absolute value of AL were measured from the corneal vertex to the center of optic nerve (Fig. 3b). We found a correlation between these two measurement methods (Fig. 3c) although the resolution of μCT (10 μm) was not as high as that of the OCT system. Interestingly, we found that the shape of the eyeball changed in only five minutes after the mouse died (Fig. 3d). This indicated that it is better to measure AL in living mice than enucleated eyeballs.

### A robust and lens power dependent myopic induction in mice with minus power lenses

**Figure 4:**
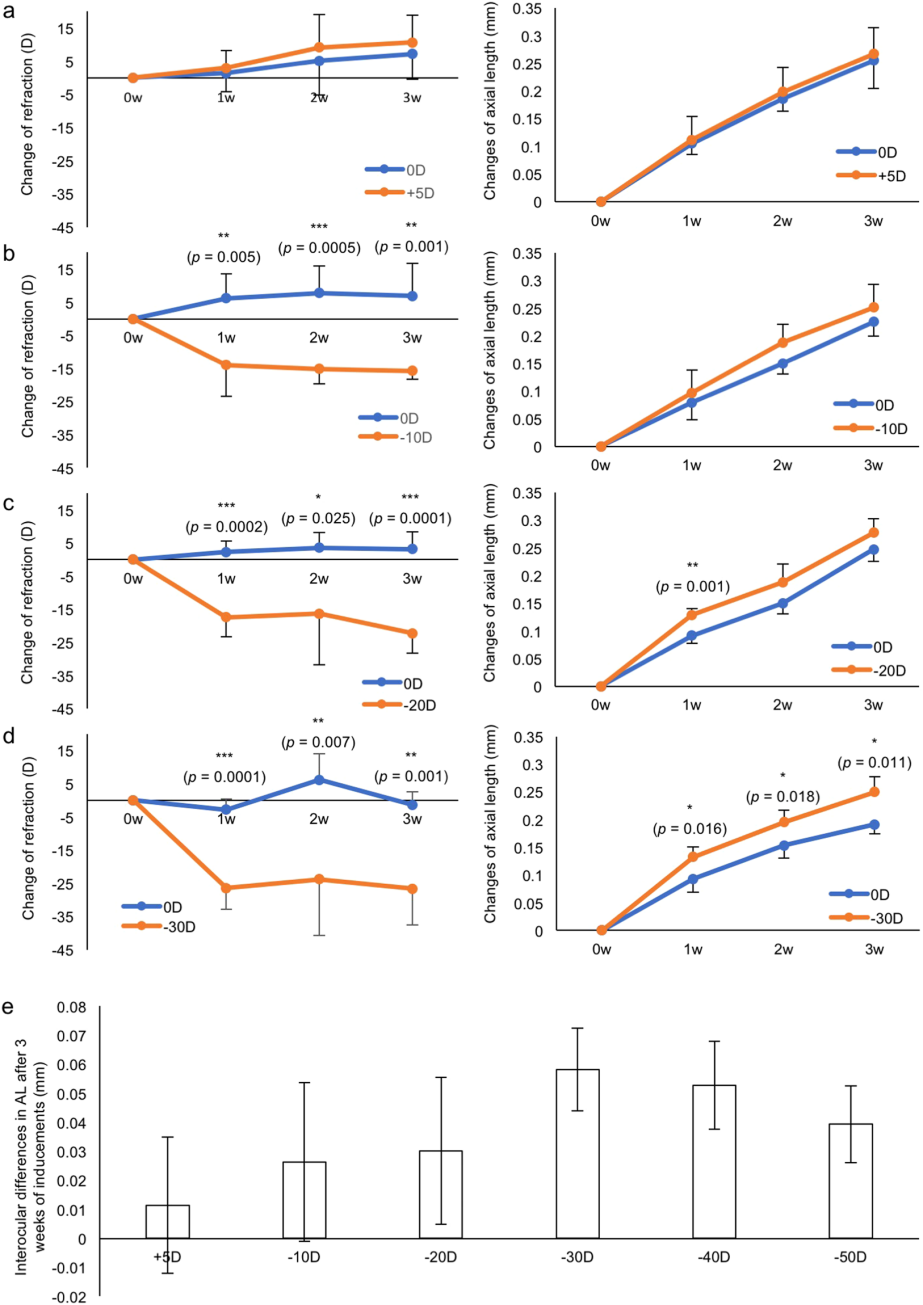
A lens power dependent myopic induction. Changes of refraction and AL during 3 weeks of lens-wearing with +5 D (**a**), −10 D (**b**), −20 D (**c**) and −30 D (**d**) are shown (n = 5 for each group). In contrast with the slowly changing AL, a sudden change can be found in refraction changes. (**e**) Interocular differences in AL after 3 weeks of inducements with +5 D, −10 D, −20 D, −30 D, −40 D, and −50 D. Note that −30 D lens induces the greatest myopic shift in AL. *p < 0.05, **p < 0.01, ***p < 0.001. Error bars indicate mean ± s.d.

We verified whether mouse eyes can adapt to the lens with different powers. Plus 5 D (Fig. 4a), −10 D (Fig. 4b), −20 D (Fig. 4c), and −30 D (Fig. 4d) lenses were fixed to the right eye side of the frame respectively, and 0 D plano lenses were fixed to the left eye side as internal controls. Changes in each part of the eye were shown in Supplementary Fig. 5. No significant change was observed in the corneal curvature (Supplementary Fig. 6). We further examined the myopic induction with −40 and −50 D lenses (Supplementary Fig. 7). After three weeks of lens wearing, a significant lens power dependency was observed in AL changes. Minus 30 D lens induced the most significant myopic shift (Fig. 4e). We also induced myopia using −30 D lens from p28 for 3 weeks. Similar myopic changes were seen in these mice although the absolute values of changes in ocular parameters were smaller compared to the induction from p21 (Supplementary Fig. 8).

### A greater myopic induction of the LIM model compared to a FDM model

**Figure 5:**
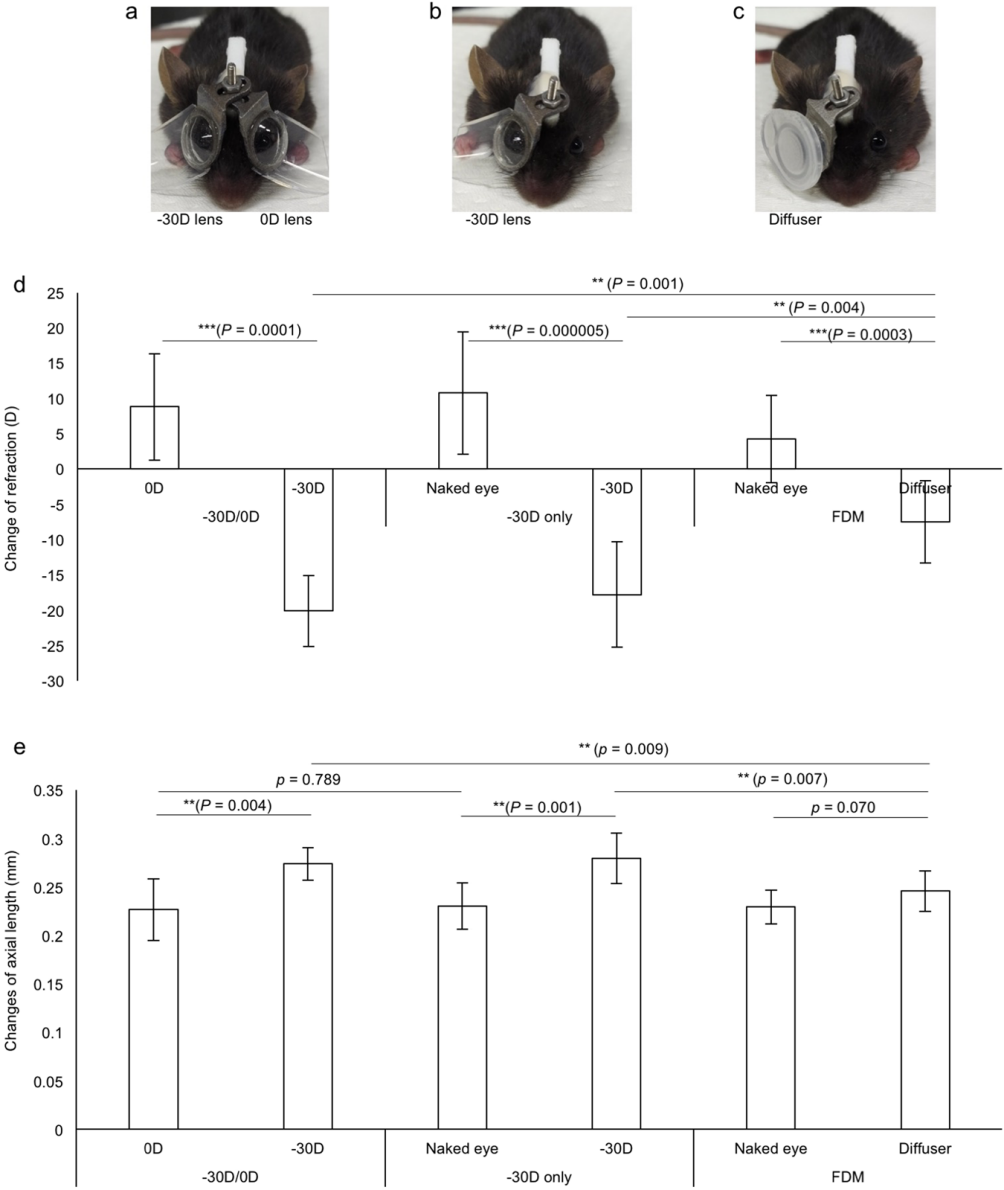
Comparison of the current LIM model with a FDM model. The phenotypes of LIM with −30 D and 0 D lenses (**a**, n = 5), LIM with −30 D lens and naked eyes (**b**, n = 8), and FDM and naked eyes (**c**, n = 10) are compared. After three weeks of inducement, all the mice in the three groups show a myopic shift in refraction (**d**) while no significant difference in AL can be found in the FDM group (**e**). LIM can induce greater myopia than FDM on changes of both refraction and AL. Wearing 0 D lenses have no detectable influence on the change of refraction and AL. **p < 0.01, ***p < 0.001. Error bars indicate mean ± s.d.

FDM has been wildly used as an experimental myopia model^17,21,36–38^. We compared the myopic phenotype between FDM and our newly designed LIM with −30 D lenses (Fig. 5a). To investigate the influence of 0 D plano lens, we also prepared one group of LIM mice with no covered lens as the control (Fig. 5b). FDM was performed with the diffuser utilizing a cap of 1.5 ml microtube (Fig. 5c). The LIM group showed a significant greater myopic shift compared to the FDM group in both the refraction (Fig. 5d) and the AL changes (Fig. 5e), while no significant difference was observed between the naked eye and 0 D lens worn eye. These data indicated that the newly designed LIM model showed a more stable and robust phenotype compared to a conventional experimental myopia model. Changes of other ocular parameters were shown in Supplementary Fig. 9. No significant change was observed in the corneal curvature (Supplementary Fig. 10).

### Decreased visual acuity of the LIM model

**Figure 6:**
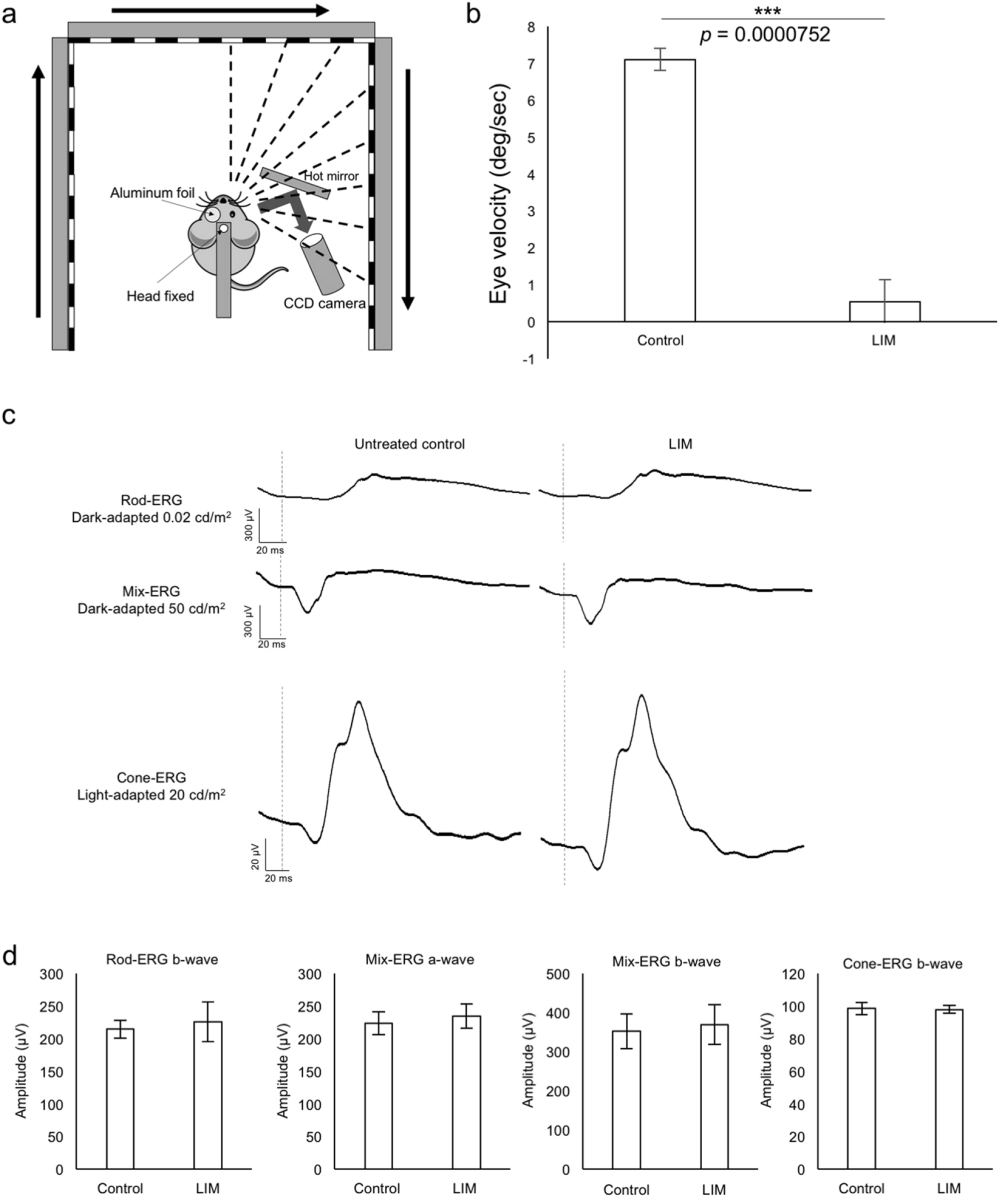
Decreased visual acuity in the mouse LIM model. (**a**) Schematic view of the OKR system. The images of the right or left eyes are captured by a CCD camera placed on the same side. During the measurement, the contralateral eyes are covered with aluminum foil. Visual stimulation is presented on three LCD monitors around the mouse whose head is fixed in the middle. The schematic view is drawn referred to the paper by Tabata H *et al*.^33^. (**b**) Eye velocities of control and myopic eyes (n = 3). Note that very little response can be detected on myopic eyes. (**c**) Representative waves of rod, mix, and cone ERG from control eyes and contralateral myopic eyes treated with −30 D lens for 3 weeks. (d) No significant difference is found in amplitude of each ERG condition (n = 3). ***p < 0.001, Error bars indicate mean ± s.d.

Besides refraction and axial length, we investigated the visual acuity of our LIM mice model. OKR tests were performed on mice with their myopic right eyes treated with −30 D lenses for 3 weeks and left eyes remained untreated as control (Fig. 6a). The lenses were taken off before the test. Tests were performed with a stimulation that induced the largest smooth eye movements in WT mice^33^ (spatial frequency: 0.125 cyc/deg, temporal frequency: 1.5 Hz). We found nearly no responses on myopic eyes while appropriate responses were detected on the control contralateral eyes (Fig. 6b). To verify the retinal function, we performed ERG tests on the same mice after the OKR tests. We confirmed that retinal function was not affected in eyes of the LIM (Fig. 6c,d). To our knowledge, this is the first report regarding decreased visual acuity detected by OKR in myopic mice.

### Application of the LIM model for ***in vivo*** drug screening

**Figure 7:**
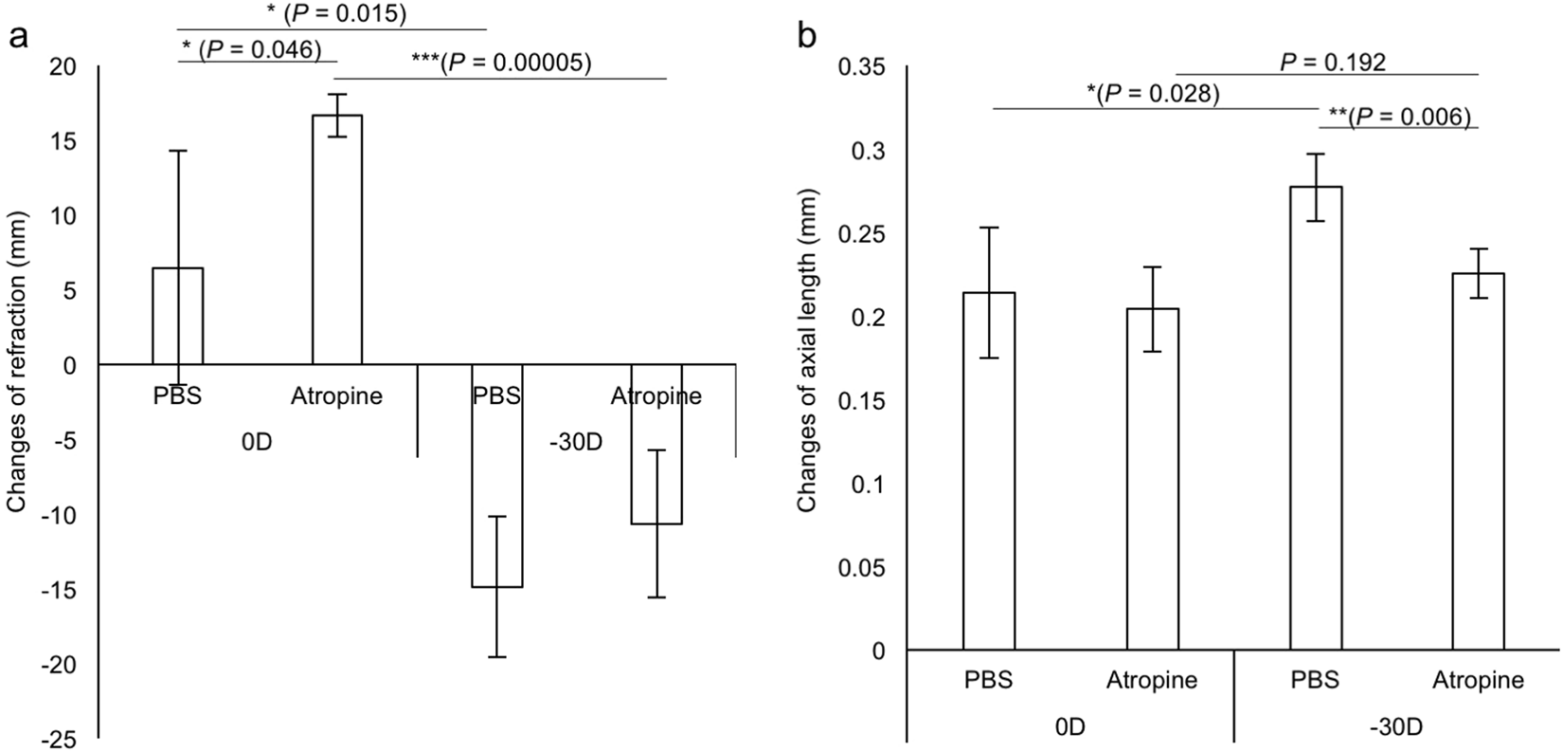
The therapeutic effect of atropine for 3 weeks on the mouse LIM model using −30 D lenses. (**a**) A hyperopic shift can be seen by tropic atropine treatment in refraction. (**b**) The myopic shift can be fully canceled by tropical atropine treatment in AL (n = 4 for each group). *p < 0.05, **p < 0.01, ***p < 0.001. Error bars indicate mean ± s.d.

Finally, we verified the LIM model as an *in vivo* myopia drug screening tool. Atropine has been confirmed as an effective anti-myopia drug in several clinical studies^35,39,40^ and animal studies^41–44^. Vehicle or 1% atropine was topically administrated as an eyedrop one time per day for three weeks for the LIM model with −30 D lenses. The slowing down of the myopic shift was apparent in both refractions (Fig. 7a) and AL (Fig. 7b) in the atropine group compared to the controls. Other ocular parameters were shown in Supplementary Fig. 11. The data indicated that the current murine model is sufficient to reproduce results of clinical studies in part, and is useful for *in vivo* drug screening model.

## Discussion

Currently, the molecular and cellular mechanism of myopia development and progression are largely unknown, which obstructs our understanding of the visual system and the development of effective anti-myopia drugs^1,26^. The deficiency of a proper myopic model has partially limited progress in research on myopia. In the current study, we described a highly robust and stable myopic mouse model which may accelerate myopic research.

Among all the myopic models, chicks may be the most popular one since the first report of FDM chicks in 1978^7^. Chicks have large eyes that allow the measurements to be done using devices designed for human beings: about 0.5 mm’s difference in AL can be achieved in one week of inducement^13^, with a large accommodative range between −10 to +20 D^45^. Because chicks are diurnal, conclusions from mice experiments may need to be tested again with chicks especially for the role of light environments^13,46^, dopamine system^47,48^ and circadian rhythm^49^ in myopia. Tree shrews also have several advantages as myopia models: they are close relatives to primates, and respond accurately to defocus in less than 2 weeks^50–52^. The differences in AL can be up to 0.12 mm which can be detected using A-scan ultrasound^10^. The difficulty in handling and breeding and the lack of fundamental biological tools limits its application in the myopia research. Utilizing non-human primates, including marmosets and rhesus monkeys, might be ideal for myopia research because they have foveae, good visual acuity, and similar body structure to humans. However, they need special care in terms of both cost and ethical issues, long duration of inducements is needed (about 20 weeks for marmosets^53,54^ and typically more than a year for rhesus monkeys^55,56^), and the data may vary in different individuals. While murine models can be used for large scale screening, non-human primates serve as a bridge between the rodents and the human beings.

Mice are widely used as animal models for biomedical research. Most importantly, gene manipulations in mice are easily achieved among various experimental animal species. To induce myopia in mice, FDM is relatively easy: suturing the eyelid or gluing the diffuser in front of the mouse eye does not require complicated further maintenance. However, the phenotype of FDM is proved to be difficult to reproduce and unstable among different studies: the change in refractive error ranges from −2 D to −9 D and the change in AL ranges from 40 *μ*m to 80 *μ*m in two to three weeks^6,19,27,30,36,57,58^, which matched well with our current findings (Fig. 5). The fluctuation may partially be due to the diffusers used in various labs differ in permeability, or the serious complications affecting the development of eyeball physically caused by eyelid suturing.

The FDM model unavoidably influences the light transmission to the eye which obstructs the intended light exposure. As the light environment has been proven to play a vital role in the onset of myopia^13,20–25,59^, LIM models which allow complete passage of the light are indispensable. To induce LIM in mice, two problems need to be solved: (1) how to affix the lens in front of the mouse eye stably while allowing it to be removed for cleaning or eye measuring, (2) how to obtain comparable measured values across experiments or even labs. To solve problem 1, we designed a simple but functional mouse frame using a three-dimensional printer and fixed it on the mouse head using a self-cure dental adhesive system (Fig. 1) which has been shown to be useful in tree shrews^32^. We referred to reported skull-mounted glasses^18,32^, and meliorated our design. Compared with a recently reported frame for mice^15^, three-dimensional printer may overcome the limitation of material processing. The design of the frame joint makes repeatable cleaning and measuring possible, and can be adapted to the growth of the mouse head. The surgery can be finished within 10 minutes by trained technicians. To solve problem 2, highly specific measuring devices along with strict measuring criteria are needed (Fig. 2). We hope the methodology described in this article can make the measured values comparable across the laboratories around the world.

Limited reports using LIM mice could be found, and the phenotypes varied a lot just as FDM. Tkatchenko’s group observed a −15 D of interocular difference on refraction and 60 *μ*m of interocular difference in AL after three weeks wearing of −25 D lenses in a 24-hour light condition^14^. Barathi’s group used −15 D lenses and found a −13 D difference in refraction. The change of AL reported by the same group was different in scale (more than 300 *μ*m) than other groups, which seems to be unnatural and might be the results of different measurement devices or the influence of enucleation^9,44^. Our team for the first time observed an apparent lens power dependency of the myopic shift in the current murine model. −30 D lenses with three weeks’ duration induced the most effective myopic shift in the present study, which can be employed as a standard protocol (Fig. 4). The use of 0 D lenses showed no significant difference on eye parameters compared to the naked eye, which can serve as a strict internal control (Fig. 5). As consistent as other reports^6,19^, the change in refraction was much faster and more drastic than the change of AL. While no significant change was found in corneal curvatures (Supplementary Figs 6 and 10), some other factors such as the position, curvature, and composition of the crystalline lens may contribute to the change of refraction prior to the AL change.

By changing the open angle of the frame, inducement can start as soon as the mouse weaning at about p21. A later starting time point such as p28 could also induce a significant myopic shift in the refraction while the change in AL was relatively small (Supplementary Fig. 8). We also performed a FDM experiment showing that the change of AL was not statistically significant in three-week inducement although a certain significant change in refraction was obtained (Fig. 5). According to the previous reports^16,19,21,31,36,37^, the phenotypes of FDM vary across the labs, but many of these showed small AL change consistent with the current result.

We also compared the OCT measurement with μCT system which has been widely used in other research areas such as the blood vessel and bone^60–62^. The resolution (10 μm) was not high enough to detect the change in AL although a correlation with OCT measurement was confirmed (Fig. 3). μCT could show the whole eyeball shape relatively quickly compared with MRI^14^. This method will be useful to detect vertical and horizontal eyeball change in addition to AL once the resolution can be increased. Interestingly, we found that the mouse eye shrinks only 5 minutes after death indicating that the measurement of AL by Vernier caliper or tissue sections will be less accurate than living measurements.

Refraction and axial length have been commonly measured to evaluate the state of myopic mouse models, while neither can directly identify the visual response of the mice. To investigate whether the visual acuity decreases in myopic mice similar to human beings, we performed OKR on mice with the −30 D lens induction for 3 weeks. Myopic eyes showed a significant decreased response compared to controls on visual stimuli (Fig. 6a,b and Supplementary Video 1) whereas retinal function was not affected (Fig. 6c,d). These data indicated that the LIM mice did not recognize visual stimulations without corrections just as myopic human beings. For future studies, visual response recoveries by minus lens wearing and behavior tests would be performed once these examinations are technically ready.

The newly designed skull-mounted eyeglass can induce the myopic state in mice effectively and stably. We are also investigating the methods to obtain repeatable measured value with existing devices. The therapeutic effect of atropine was reproduced in our model (Fig. 7) suggesting that the model mimics human myopia progression in part. Utilizing the current model, we believe that fundamental molecular and cellular mechanism will be revealed and mechanism-based myopia control approach will be developed in the near future.

## Methods

### Mice

All procedures were approved by the Ethics Committee on Animal Research of the Keio University School of Medicine adhered to the ARVO Statement for the Use of Animals in Ophthalmic and Vision Research, the Institutional Guidelines on Animal Experimentation at Keio University, and the Animal Research: Reporting of *In Vivo* Experiments (ARRIVE) guidelines for the use of animals in research. C57BL/6 J mice were obtained from CLEA Japan, Inc. Five wild type adult mice without any treatment were used to compare the measurements of AL using μCT and SD-OCT. For lens power dependency experiments, five p21 mice were used for each lens power. For the comparison of LIM with FDM, five p21 mice were worn with 0 D lenses and −30 D lenses, eight p21 mice were worn with −30 D only and ten p21 mice were worn diffusers only. For the atropine treatment experiment, four p21 mice worn 0 D and −30 D lenses were treated with atropine in both eyes and four p21 mice were treated with PBS in both eyes as control. For the OKR experiment, three mice worn −30 D lenses on their right eyes for three weeks started from p21 were used. After the measurement of the OKR, the same three mice were put into general anesthesia for the ERG measurements. All mice were fed with normal chew and water ad lib. Four or five mice with or without the frame were kept in one cage with approximately 50 lux background fluorescent lamp light for 12 h from 8:00 am to 8:00 pm.

### The design, output, and the setup of the mouse frame

The sketch of the design of the mouse frame was given to a third-party company to convert into three ‐ dimensional data and output with titanium for frame bodies or nylon for sticks using a three ‐ dimensional printer. Screws, nuts, washers and artificial fingernail tips were bought from retail tool stores. Lenses customized for mice were made from polymethyl methacrylate (Rainbow Optical Laboratory Co., Ltd, Tokyo, Japan). For FDM experiments, the cap of 1.5 ml microtube (AS ONE Corporation, Osaka, Japan) was used as the diffuser.

To affix the frame on the mouse’s head, the mouse was put under general anesthesia using a combination of midazolam (Sandoz K.K., Tokyo, Japan), medetomidine (Domitor®, Orion Corporation, Espoo, Finland,) and butorphanol tartrate (Meiji Seika Pharma Co., Ltd., Tokyo, Japan), MMB for short. The scalp was cut to expose a 0.8 square meters’ area of the skull, and the periosteum was removed by etching liquid. The stick with a screw was attached onto the skull directly using a self-cure dental adhesive system. After the mouse completely recovered from the anesthesia, the frame was attached and the inducement began. The lenses or diffusers were removed for cleaning at least twice a week.

### The refraction and corneal curvature measurements

An infrared photorefractor (Steinbeis Transfer Center, Germany) was used to measure the refractive state^6,63^. The mouse eye was instilled by Tropicamide, Penylephrine Hydrochloride solution (Mydrin-P ophthalmic solution, Santen Pharmaceutical Co., Ltd) 5 minutes before the measurement to ensure mydriasis and cycloplegia. After the injection of MMB to induce general anesthesia, 99 measurements were taken along the optical axis for one eye and the averages were recorded as the refraction. The way to obtain stable results was described precisely in the results.

Another infrared photorefractor (Steinbeis Transfer Center, Germany) was used to measure the corneal curvature according to previous reports^63^. After the injection of MMB to induce general anesthesia, eight infrared LEDs placed as a disc lit the mouse cornea, and the reflected light was detected with an infrared camera. The diameter of the circle made by the reflected light on the mouse cornea was taken as the corneal curvature.

### The measurement of ALs and other eye parameters using SD-OCT

The AL of the eye was analyzed by a SD-OCT system (Envisu R4310, Leica, Germany) tuned for mice. The mouse under general anesthesia was put into the tube that can move the position of the mouse precisely. After capturing the whole eyeball, the mouse was kept at the same position for an extra shot of the retina to confirm the position of the slice of the AL to be near the optical nerve. The relationship between the measured value and the relative position of the slice with the optical nerve was described in the results.

### The whole eyeball scan using μCT

A μCT system (Rigaku, Japan) was tested for the measurement of the mouse eye structure. Before the CT scan, the mouse was put under general anesthesia and performed with one SD-OCT scan for cooperation. Then the jugular vein was exposed for intubation. 0.1 ml/10 g Iohexol (OMNIPAQUE 300, GE Healthcare Inc.) was injected into jugular vein for contrast enhancement. The images were saved as dicom format for further analysis using Osirix^®^ (Newton Graphics, Inc., Sapporo, Japan).

### ERG

Scotopic and photopic ERGs were recorded using a Ganzfeld dome, an acquisition system (PuREC, MAYO Corporation, Japan), and an LED stimulators. Following overnight dark adaptation, mice were anesthetized using MMB under dim red light. Pupils were dilated with tropicamide and phenylephrine hydrochloride (Santen Pharmaceutical Co., Ltd., Japan). The active electrodes were recorded with contact electrodes and the reference electrode was placed in the mouth. A clipping electrode to the tail served as a ground. The ERG responses were obtained from both eyes of each animal. Rod ERGs were recorded under dark adaptation with 0.02 cd/m² stimulus intensity, and 4 responses were recorded and computer-averaged. Mixed rod and cone responses (Mix ERGs) were obtained by a white flash 50 cd/m² stimulus. To assess photopic ERGs, the mice were adapted for 5 minutes under a white background (30 cd/m²). Cone ERGs were recorded with 20 cd/m² stimulus, and 32 individual responses were averaged. The low pass of the amplifiers was set at 30 Hz.

### OKR recording system

Protocols for eye movement recording and visual stimulation were previously described^33,64^. Eye movements were recorded from both eyes of each animal separately. During the recording, the contralateral eye was covered by aluminum foil. Head of the mouse was fixed to an experimental steel board by the head mounted stick for the LIM lens frames. The reflected images through a hot mirror (43957-J, Edmund) were recorded using an inflared CCD camera (BS-GV200, Libraly inc, Tokyo, Japan). The images of the eye movements were processed and analyzed using a software (Move-tr/2D, Libraly inc, Tokyo, Japan). The sampling rate of the image was 200 Hz. The center of the pupil was detected on software. We calculated the speed of the eye movements on two- dimensional images and converted them to angular speeds using the AL of each eye. To obtain the maximal speed of eye movement speed of wild type C57BL/6 J mice, the spatial frequency was set as 0.125 cycle/degree and temporal frequency of the visual stimulus as 1.5 Hz, respectively as previously described^33^. The motion onset delay (MOD) was set as 333 msec. Continuing the MOD, sinusoidal grating started to move clockwise in 5 sec. The intervals of visual stimulus were 60 sec. Eye movements were recorded three times for each to exclude shaking images due to excessive body movements. Average velocities of the eye movements were calculated in the slow speed phase of their nystagmus.

### Administration of topical 1% atropine eye drop

The powder of atropine sulfate monohydrate was bought from Wako Pure Chemical Industries, Ltd. 100 mg powder was added into 10 ml sterilized PBS to prepare 1% atropine eye drop. The mouse with 0 D lens in front of the left eye and −30 D lens in front of the right eye were instilled with 1% atropine or vehicle (pure PBS) one drop a day. Before the instillation, lenses were removed and cleaned.

### Statistical analyses

*T*-test was used to analyze statistical significances of all the data in this paper and the degree of freedom was 2. *P* value below 0.05 was considered significant. All data are presented as mean ± standard deviation.

### Data availability

The data sets generated during and/or analyzed during the current study are available from the corresponding authors on reasonable request.

## Electronic supplementary material

Supplementary information

Supplementary video S1
