# Supplementary material for: A highly efficient murine model of experimental myopia
Source: Sci Rep. 2018 Feb 1;8:2026. doi: 10.1038/s41598-018-20272-w (PMC5794929; doi:10.1038/s41598-018-20272-w)

Supplementary Information

A highly efficient murine model of experimental myopia

Xiaoyan Jiang[1,2], Toshihide Kurihara[1,2*], Hiromitsu Kunimi[1,2], Maki Miyauchi[1,2], Shin-ichi Ikeda[1,2], Kiwako Mori[1,2], Kinya Tsubota[1], Hidemasa Torii[1,2] and Kazuo Tsubota[2*]

[1]Laboratory of Photobiology, [2]Department of Ophthalmology, Keio University School of Medicine, Tokyo, Japan

Supplementary Figure Legends

Supplementary Figure 1. Trials and errors on the designing of the frame.

Five generations of frames designed were shown in chronological order. The first generation of the frame was made of nylon, which was too fragile for three-week wearing. In the second generation, we changed the material to titanium. Then, we separated the frame into two parts which allowed the angle of the frame to be adjusted to keep the eye in the middle of the frame as the mouse grew in the third generation. We further added artificial fingernail tips to prevent the mouse from scratching the lens in the fourth generation. This fifth generation with the enlarged angle of the nail tip was the final version currently used.

Supplementary Figure 2. An extreme example to show the importance of gaze control in refraction measurements.

In this mouse, only 4 degrees of change in x axial (regions outlined in blue lines) resulted in more than 30 diopters change in refraction value (regions outlined in orange lines).

Supplementary Figure 3. The best position of the refraction measurement can be achieved relatively easily with the help of the tube designed for SD-OCT.

The figure shows an example of the arrangement of the photorefractor for the refraction measurement (a), the photorefractor for corneal the curvature measurement (b), the lens of SD-OCT (c), and the tube for adjusting the position of the mouse (d).

Supplementary Figure 4. The investigation of the proper position of the boundary of the retinal side for SD-OCT measurements.

(a) We compared the AL value with three positions: from the corneal vertex to the center of the optic nerve (position 1), to about half diameter of the optic papilla away from the center (position 2), and to one diameter of the optic papilla away from the center (position 3). The retinal images are shown above and the slice for AL measurements taken in the same position is shown below. (b) The comparison of AL values measured in positions 1, 2, and 3 in ten mice, respectively. Except for some mice, the values tended to be smaller with the segment away from the optical nerve. (c) Schematic views of the three positions are shown in this figure. Orange cylinders indicated for the optical nerve. Arrows indicated for ALs measured by SD-OCT. For position 1, the outer borderline of retina was not clear enough for precise measurements, while position 3 had no marker to ensure every measurement was in the same place. For position 2, the borderline can be recognized while the haze can ensure that every measurement was not too far from the optical nerve. We eventually defined the AL of mice as the length from corneal vertex to position 2 in this study.

Supplementary Figure 5. Changes of ocular parameters induced by several different power lenses.

Changes in each part of the eye after 3 weeks of +5 D (a), -10 D (b), -20 D (c), and -30 D (d) lens-wearing started from p21. n = 5. *$p < 0.05$. Error bars indicate mean plus s.d. CT: corneal thickness. ACD: anterior chamber depth. LT: lens thickness. VCD: vitreous chamber depth. RT: retinal thickness.

Supplementary Figure 6. Changes of the cornea curvature induced by several different power lenses.

No significant change in cornea curvature after 3 weeks of +5 D (a), -10 D (b), -20 D (c), and -30 D (d) lens-wearing started from p21. n = 5. Error bars indicate mean plus s.d.

Supplementary Figure 7. Changes of ocular parameters induced by -40 D and -50 D lenses.

Changes of refraction (a), axial length (b), corneal curvature (c), corneal thickness (d), anterior chamber depth (e), lens thickness (f), vitreous chamber depth + retinal thickness (g) in -40 D group and changes of refraction (h), axial length (i), corneal curvature (j), corneal thickness (k), anterior chamber depth (l), lens thickness (m), vitreous chamber depth + retinal thickness (n) in -50 D group after 3 weeks of lens-wearing started from p21. n = 5, respectively. *$p < 0.05$, **$p < 0.01$, ***$p < 0.001$. Error bars indicate mean plus s.d.

Supplementary Figure 8. Changes of ocular parameters induced by -30 D lens started from p28.

(a) Changes of refraction. (b) Changes of AL. (c) Changes of corneal thickness. (d) Changes of anterior chamber depth. (e) Changes of lens thickness. (f) Changes of vitreous chamber depth + retinal thickness. Similar changes can be observed with the group started from p21 while the absolute values were smaller. n = 5. *$p < 0.05$, **$p < 0.01$, ***$p < 0.001$. Error bars indicate mean plus s.d.

Supplementary Figure 9. Comparison of the current LIM model with a FDM model with the ocular parameter changes.

Changes of each ocular parameter in LIM with -30 D and 0 D lenses group (a), LIM with -30 D lens and naked eye group (b), and FDM and naked eye group (c). *$p < 0.05$, **$p < 0.01$. Error bars indicate mean plus s.d. CT: corneal thickness. ACD: anterior chamber depth. LT: lens thickness. VCD: vitreous chamber depth. RT: retinal thickness.

Supplementary Figure 10. Comparison of the current LIM model with a FDM model with the corneal curvature changes

No significant change in corneal curvature can be found in LIM with -30 D and 0 D lenses groups (a), LIM with -30 D lens and naked eye group (b), and FDM and naked eye group (c). *$p < 0.05$, **$p < 0.01$. Error bars indicate mean plus s.d.

Supplementary Figure 11. Changes of ocular parameters by administration of the topical atropine eye drops.

Changes in the corneal thickness (a), the anterior chamber depth (b), the lens thickness (c), the vitreous chamber depth and the retinal thickness (d), and the corneal curvature (e) of the eyes within 3 weeks treatment of topical 1% atropine eye drops compared with PBS in the LIM model using -30 D lenses.

Supplementary Video 1. Optokinetic responses were diminished in the murine model of LIM.

Supplementary Figure 1

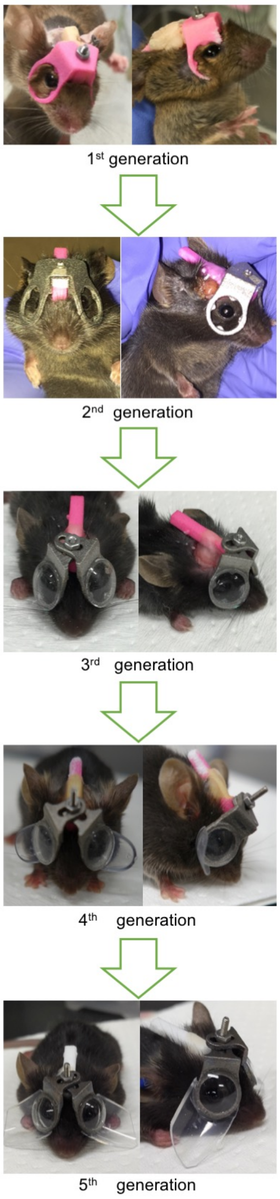

Supplementary Figure 2

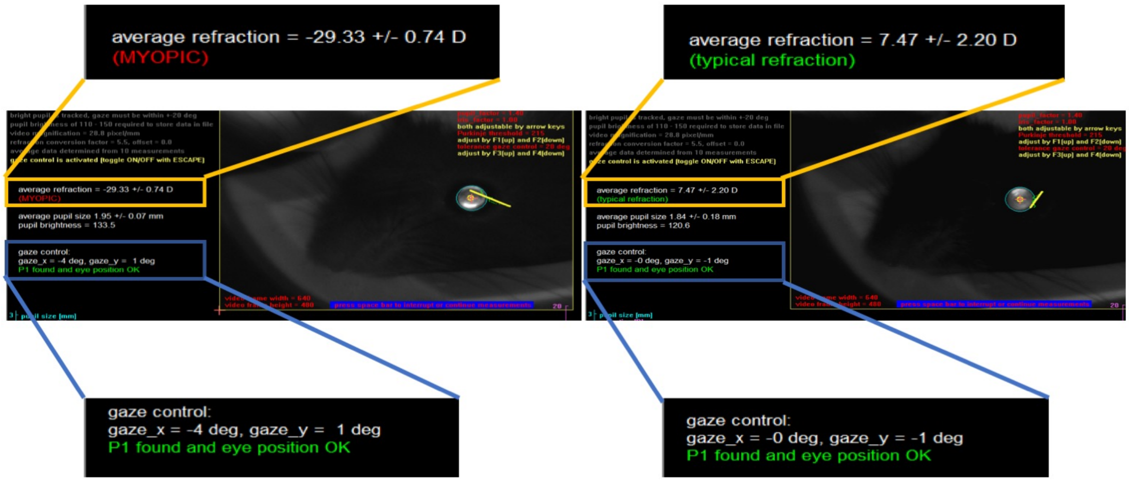

Supplementary Figure 3

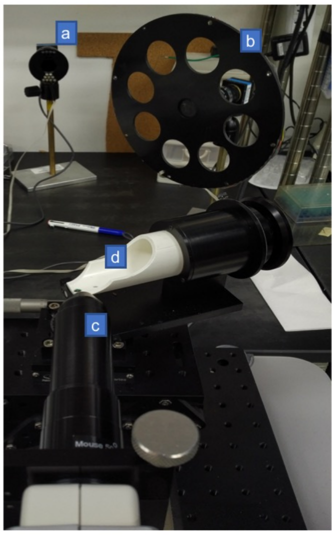

Supplementary Figure 4

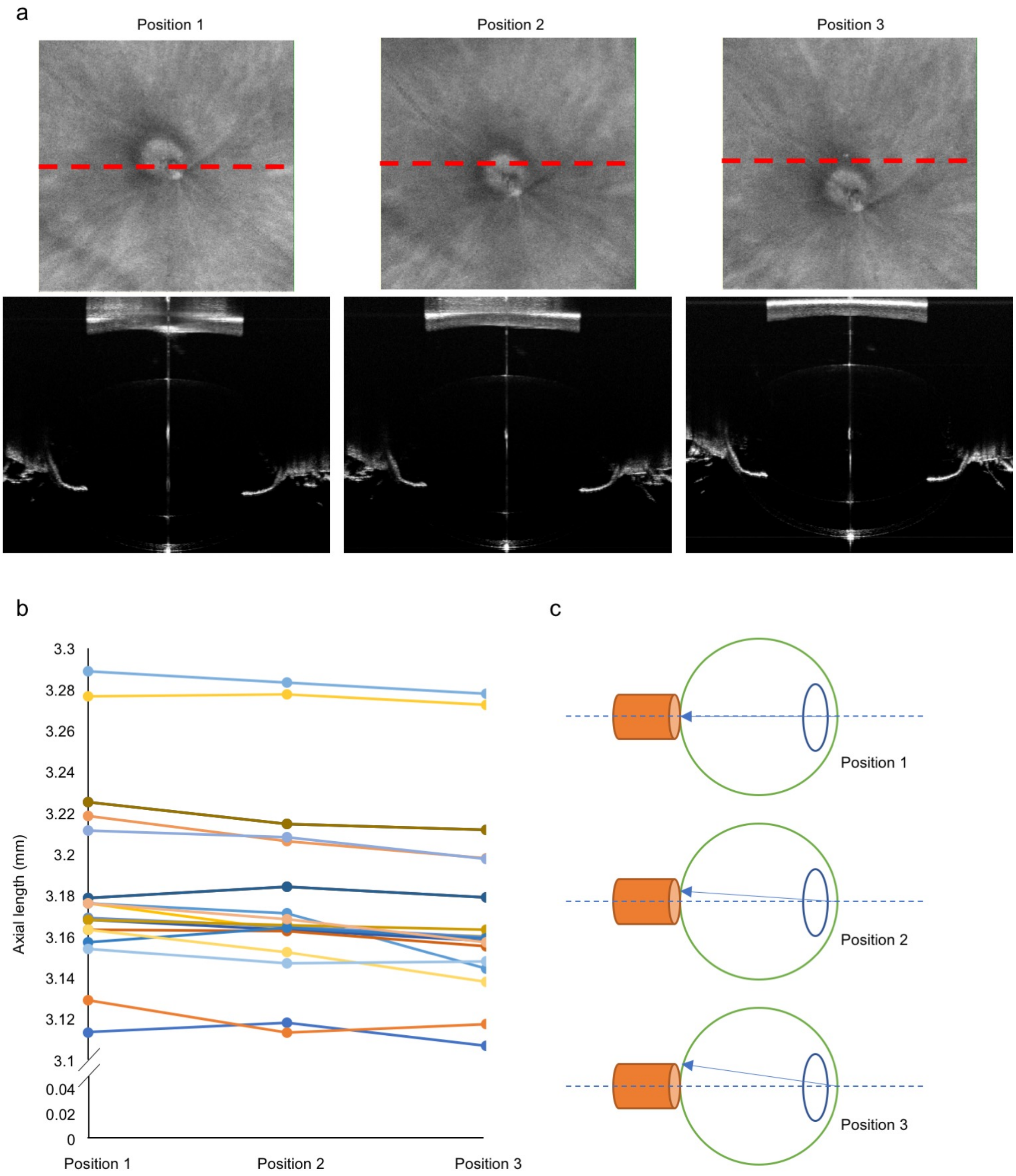

Supplementary Figure 5

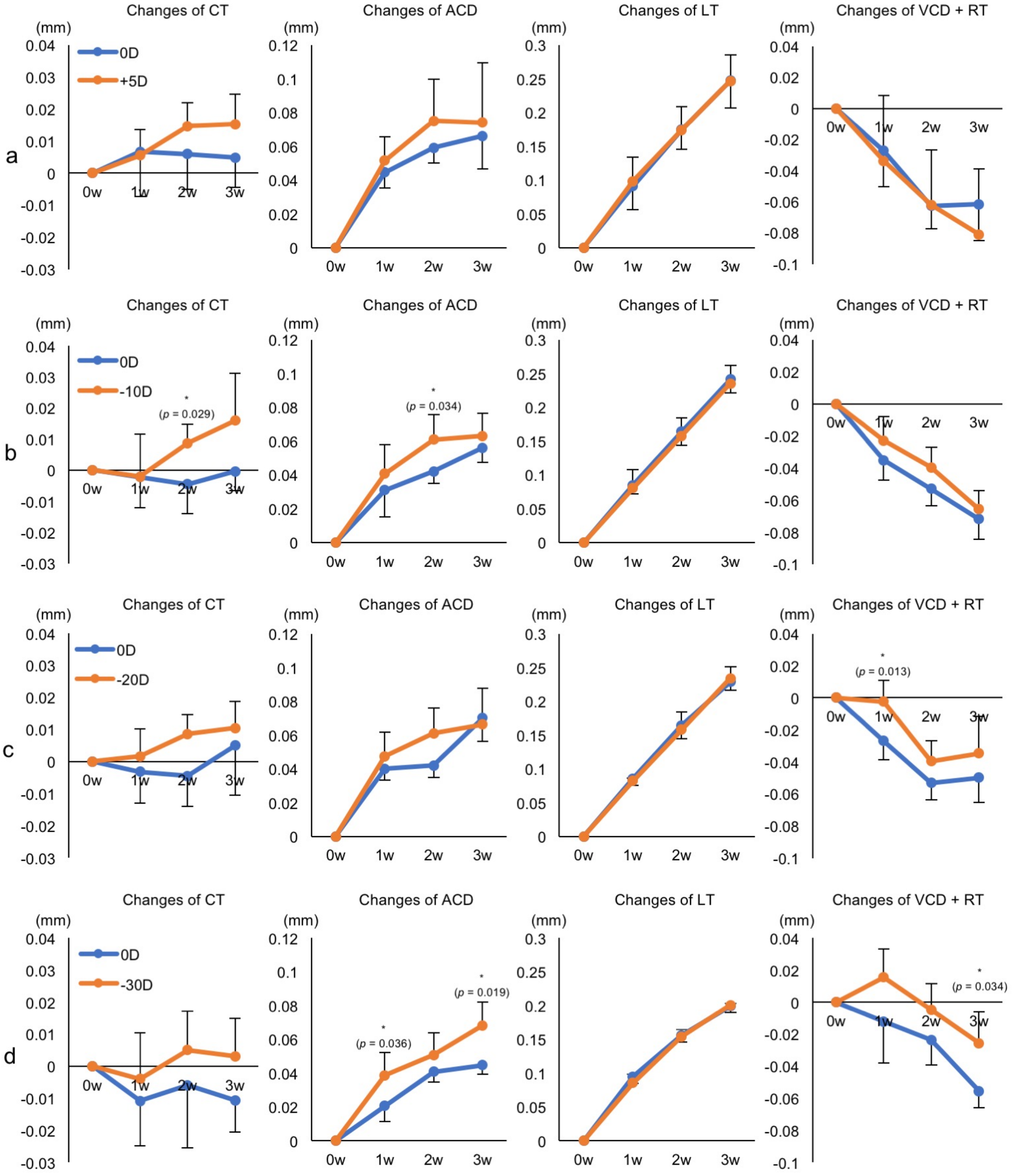

Supplementary Figure 6

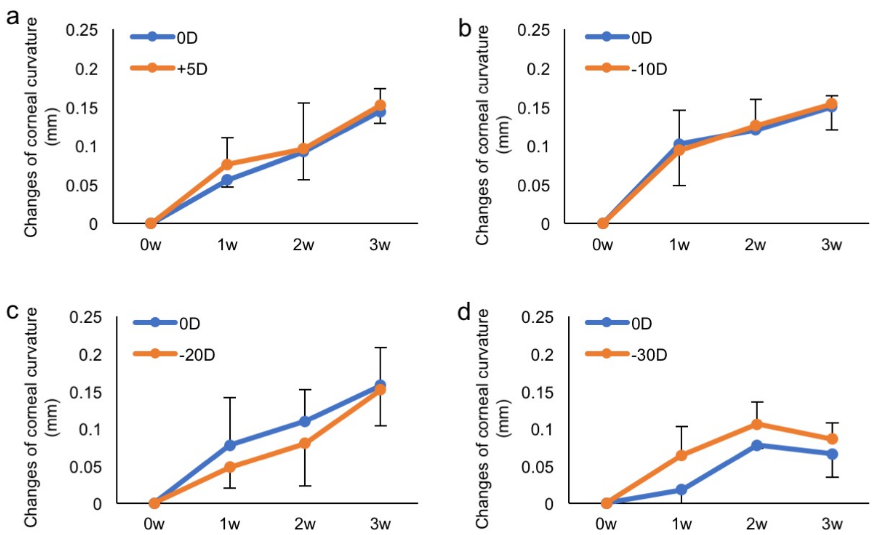

Supplementary Figure 7

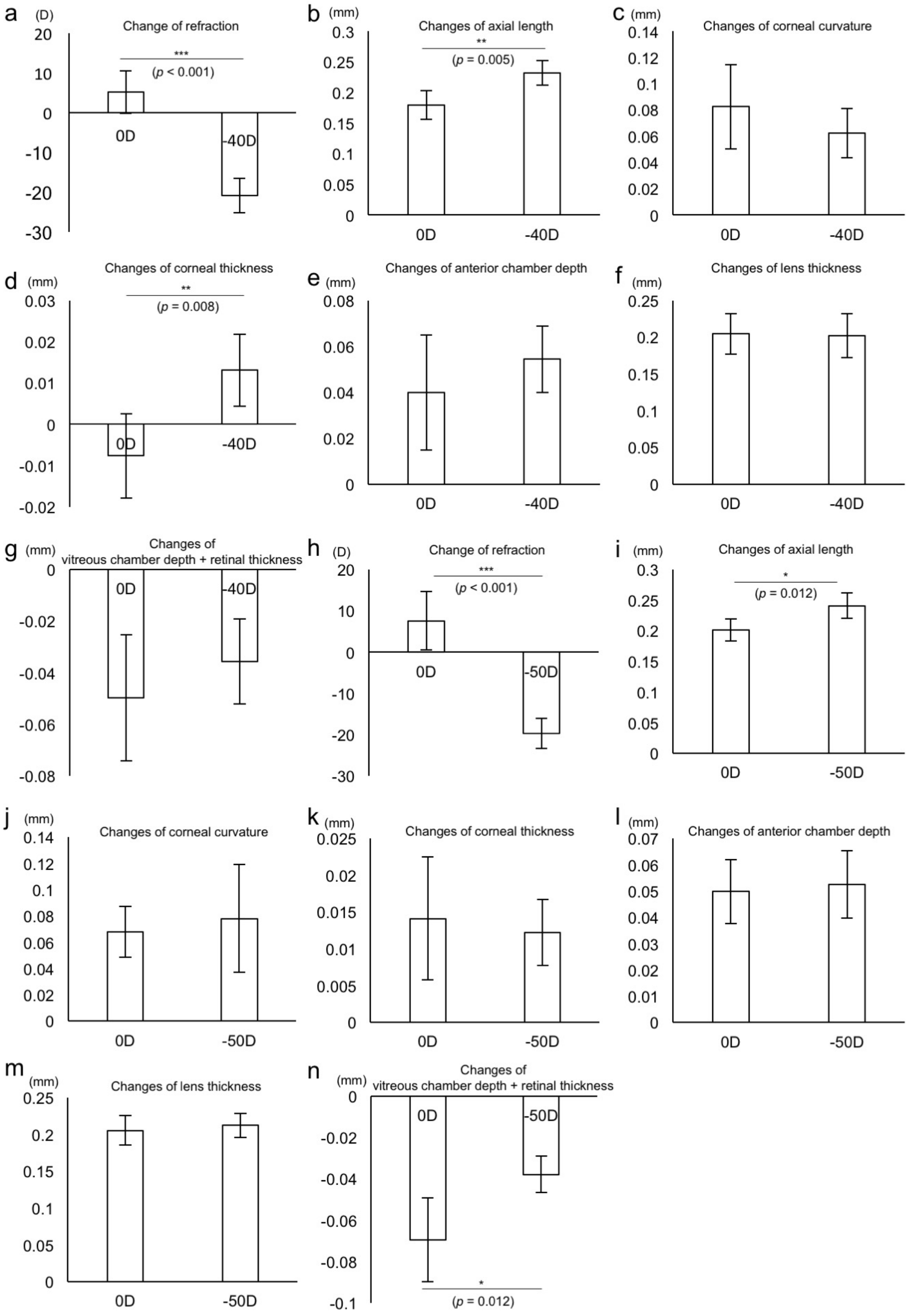

Supplementary Figure 8

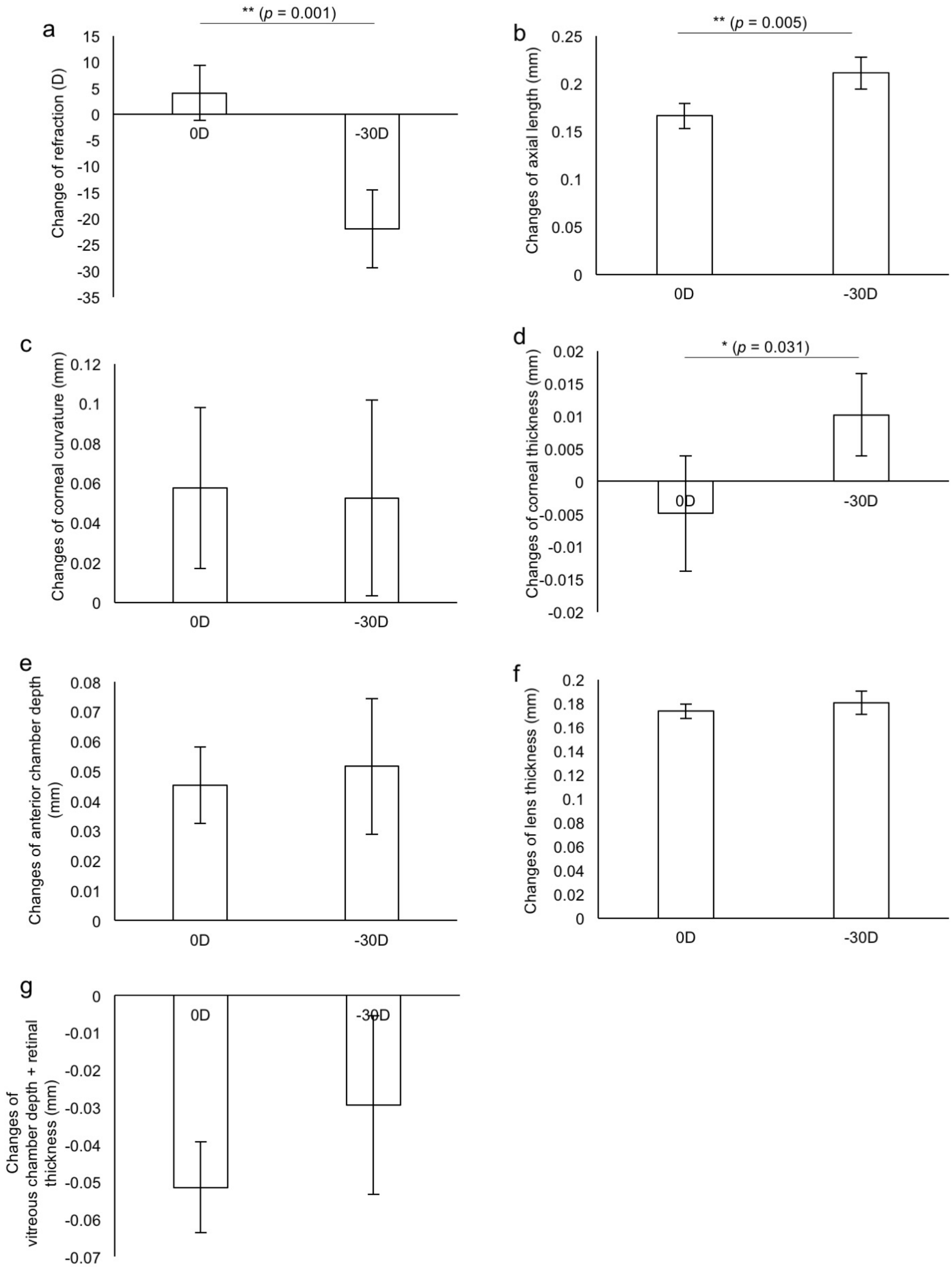

Supplementary Figure 9

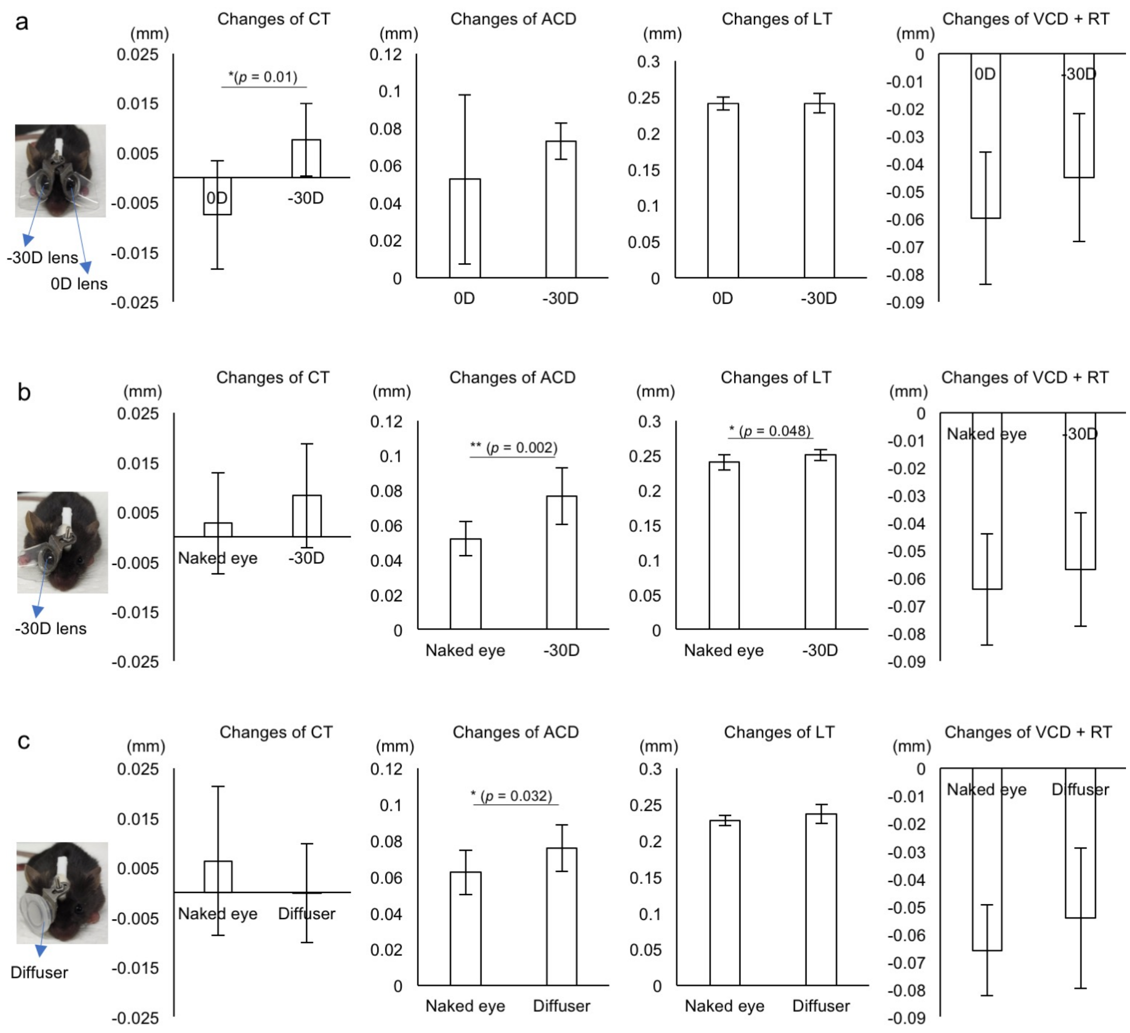

Supplementary Figure 10

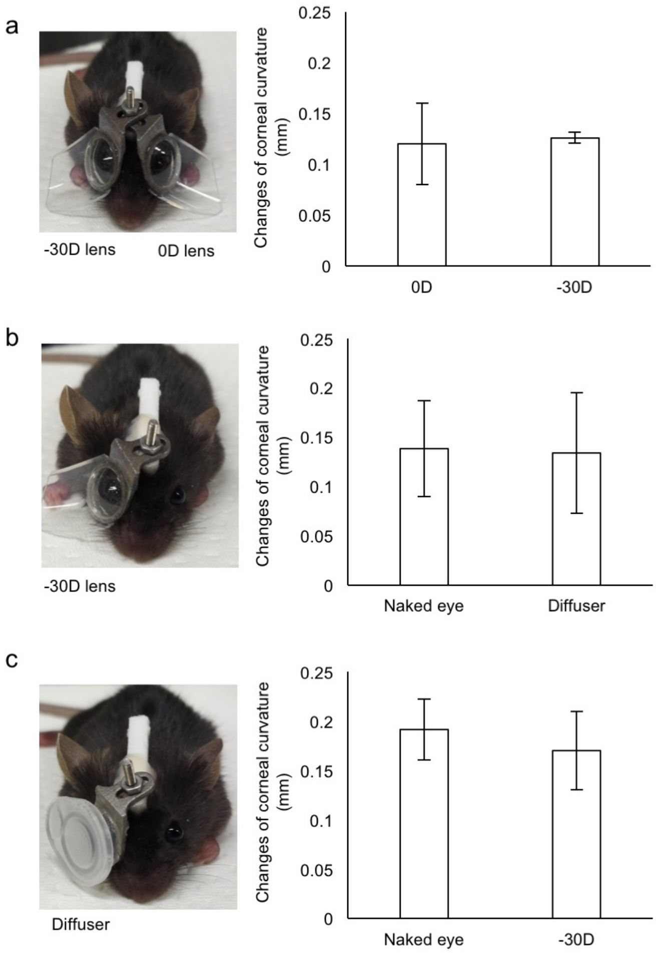

Supplementary Figure 11

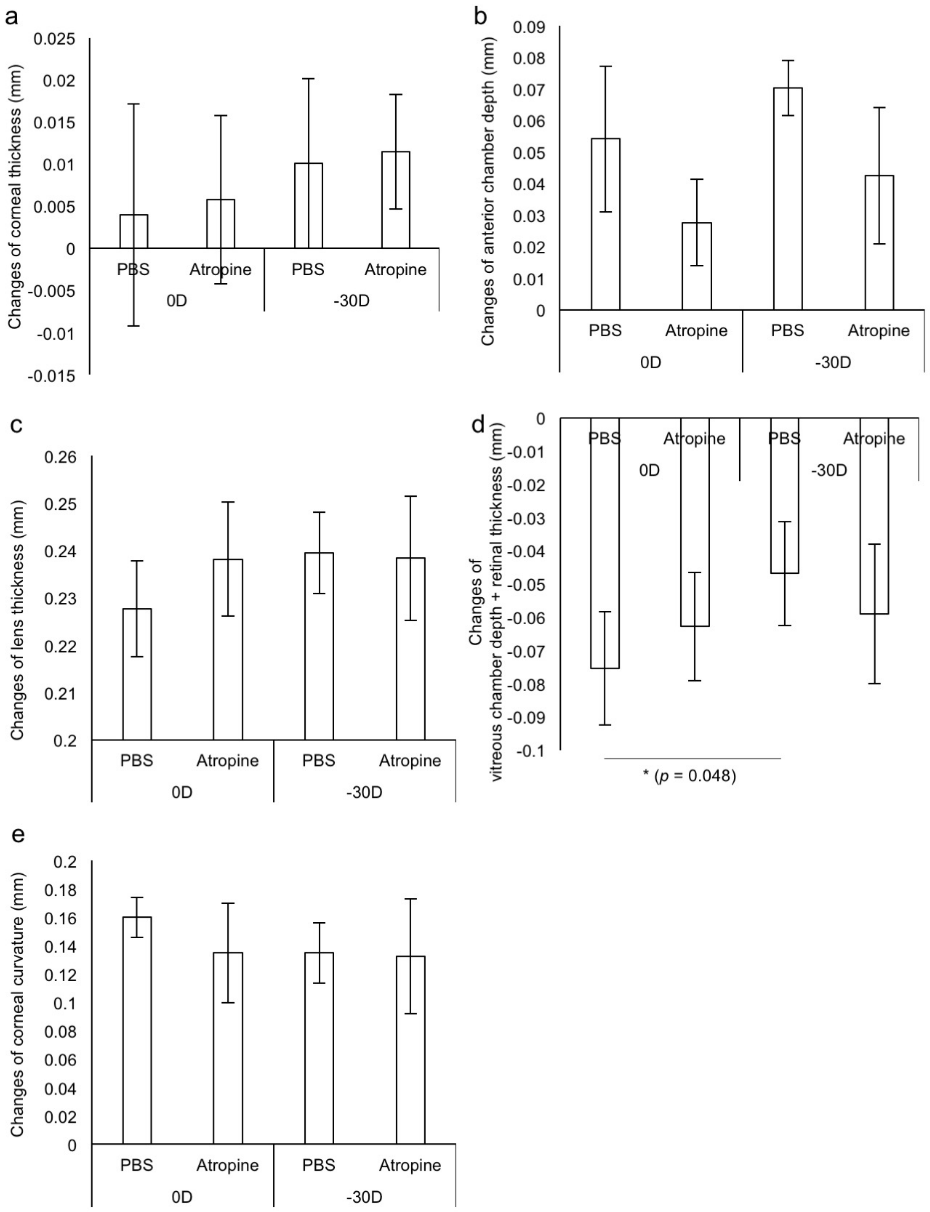

Supplement: Supplementary file 1 — Supplementary information [file 41598_2018_20272_MOESM1_ESM.pdf]
